# Non-Enzymatic Glucose Sensing Using Carbon Quantum Dots Decorated with Copper Oxide Nanoparticles

**DOI:** 10.3390/s16101720

**Published:** 2016-10-18

**Authors:** Houcem Maaoui, Florina Teodoresu, Qian Wang, Guo-Hui Pan, Ahmed Addad, Radhouane Chtourou, Sabine Szunerits, Rabah Boukherroub

**Affiliations:** 1 Institut d’Electronique, de Microélectronique et de Nanotechnologie (IEMN), UMR CNRS8520, Université Lille 1, Avenue Poincaré-BP 60069, 59652 Villeneuve d’Ascq, France; houcemmaaoui@gmail.com (H.M.); fteodorescu@icf.ro (F.T.); qian1.wang@ed.univ-lille1.fr (Q.W.); sabine.szunerits@univ-lille1.fr (S.S.); 2 Département de Physique, Faculté des Sciences de Tunis, Université Tunis-El Manar, 2092 Tunis, Tunisia; radhouane.chtourou@inrst.rnrt.tn; 3 State Key Laboratory of Luminescence and Applications, Changchun Institute of Optics, Fine Mechanics and Physics, Chinese Academy of Sciences, 3888 Dong Nanhu Road, 130033 Changchun, China; pangh@ciomp.ac.cn; 4 Unité Matériaux et transformations (UMET), UMR CNRS 8207, Université Lille1, Cité Scientifique, 59655 Villeneuve d’Ascq, France; ahmed.addad@univ-lille1.fr

**Keywords:** carbon quantum dots, copper oxide nanoparticles, glucose sensing, non-enzymatic, serum, electrochemistry

## Abstract

Perturbations in glucose homeostasis is critical for human health, as hyperglycemia (defining diabetes) leads to premature death caused by macrovascular and microvascular complications. However, the simple and accurate detection of glucose in the blood at low cost remains a challenging task, although it is of great importance for the diagnosis and therapy of diabetic patients. In this work, carbon quantum dots decorated with copper oxide nanostructures (CQDs/Cu_2_O) are prepared by a simple hydrothermal approach, and their potential for electrochemical non-enzymatic glucose sensing is evaluated. The proposed sensor exhibits excellent electrocatalytic activity towards glucose oxidation in alkaline solutions. The glucose sensor is characterized by a wide concentration range from 6 µM to 6 mM, a sensitivity of 2.9 ± 0.2 µA·µM^−1^·cm^−2^, and a detection limit of 6 µM at a signal-to-noise ratio S/N = 3. The sensors are successfully applied for glucose determination in human serum samples, demonstrating that the CQDs/Cu_2_O-based glucose sensor satisfies the requirements of complex sample detection with adapted potential for therapeutic diagnostics.

## 1. Introduction

Carbon quantum dots (CQDs) have attracted much attention in recent years, due to their outstanding optical and electronic properties [1,2]. Their excellent photoluminescence properties and tunable fluorescence emission, sensitive not only to the size of the carbon particles, but also to the presence of different analytes (e.g., metal ions, anions) have made CQDs widely used as fluorescent labels as well as for chemical sensing applications [1]. Their high stability under strongly basic and acidic conditions and good electrical conductivity have also made them interesting electrocatalytic materials for energy-related applications. Here, oxygen-reduction (ORR) and evolution (OER) reactions are at the center of interest, with the aim of improving the sluggish kinetics and high over-potential required to drive oxygen evolution [3,4]. Surprisingly, reports on using CQDs in electrochemical-based sensing applications are still rare [5,6,7]. CQDs were used in these reports either to promote direct electron transfer between a biomolecule (e.g., hemoglobin) and the electrode surface [5], or for sensing of anticancer drugs such as etoposide [6]. 

One molecule that is challenging and important to monitor is glucose. An increase in glucose levels is critical for human health, as hyperglycemia (defining diabetes) leads to premature death caused by macrovascular and microvascular complications. The accurate detection of glucose in blood is thus of great importance for the diagnosis and therapy of diabetic patients. 

Phenylboronic acid-modified CQDs have been proposed by several groups as a fluorescence-based glucose sensing system [8,9,10]. A glucose sensing system consisting of fluorescent B-doped CQDs in combination with glucose oxidase was recently developed by Shan et al. [11] as an enzyme-based glucose sensing system. However, the use of CQDs for non-enzymatic glucose sensing has not yet been investigated. Glucose oxidase-based sensors suffer greatly from the poor stability of the enzyme over time, considerably decreasing the shelf-time of these glucose sensors. The development of non-enzymatic sensors [12] based on the direct oxidation of glucose has shown several advantages, such as high sensitivity, good selectivity, and good life shell times over both fluorescence- and enzymatic-based sensors [13,14,15,16].

Copper-based electrodes represent an interesting class of materials for the electrocatalytic oxidation of glucose [13,14,15,16]. The electrocatalytic activity of these electrodes toward glucose oxidation in alkaline medium is believed to be attributable to the involvement of Cu^2+^ and Cu^3+^ surface species. Compared to other catalytic nanostructures based on gold or platinum, Cu is cheap and abundantly available. 

CQDs, on the other hand, are believed to promote electron transfer similar to other carbon-based nanomaterials. The combination of CQDs and octahedral Cu_2_O for non-enzymatic detection of hydrogen peroxide (H_2_O_2_) and glucose has recently been reported by Li et al. [17]. The CQDs/Cu_2_O nanocomposite was prepared using a hydrothermal method and ultrasonic treatment. The developed electrocatalyst exhibited a detection limit of 8.4 µM over a 20–4300 µm linear range.

In the present study, we synthesized Cu_2_O-decorated CQDs through a simple hydrothermal process with the aim of improving the electrocatalytic activity of the Cu_2_O nanostructures towards glucose sensing in solution and human serum, through their hybridization with CQDs. It is well-known that the hybridization of different nanoparticles on solid substrates limits their aggregation upon immersion in liquid solutions, and thus improves their electro/catalytic activity and stability. The method developed herein for the synthesis of CQDs/Cu_2_O is cost effective and obeys green chemistry principles, as it takes place in aqueous media.

## 2. Experimental Part

### 2.1. Materials 

Fructose, galactose, glucose, ascorbic acid, uric acid, dopamine, sodium hydroxide (NaOH), phenol, sulfuric acid (H_2_SO_4_), copper perchlorate (Cu(ClO_4_)_2_), alumina powder (1, 0.3, and 0.05 µm), Nafion, and isopropyl alcohol were obtained from Sigma Aldrich (St. Quentin Fallavier, France) and used as received. Human serum was kindly provided by the Centre Hospitalier Universitaire (CHU), Lille, France.

### 2.2. Synthesis of Carbon Quantum Dots

Carbon quantum dots (CQDs) were synthesized by a one-pot method at a relatively low reaction temperature. In a typical procedure, fructose (500 mM) and sodium hydroxide (500 mM) were added to 20 mL water, the solution was placed in a Teflon-lined stainless steel autoclave and heated at 50 °C for 1 h. The solution turned from colorless into brown [18]. 

### 2.3. Synthesis of Carbon Quantum Dots Loaded with Copper Oxide Nanoparticles (CQDs/Cu_2_O)

Copper perchlorate (1.35 g) was dissolved in 10 mL of previously prepared CQDs solution and heated at 80 °C for 3 h. The formed precipitate was separated by centrifugation, washed with water three times, and annealed at 120 °C overnight.

### 2.4. Synthesis of Cu_2_O

Copper perchlorate (1.35 g) was dissolved in 10 mL of sodium hydroxide (0.5 M) aqueous solution and heated at 80 °C for 3 h. The formed precipitate was separated by centrifugation, washed with water three times, and annealed at 120 °C overnight.

### 2.5. Preparation of CQDs/Cu_2_O-Modified Electrode

A bare glassy carbon electrode (GCE), polished before each experiment with alumina powder (1, 0.3, and 0.05 µm), was coated by drop casting 4 µL of CQDs/Cu_2_O aqueous solution (1 mg/mL) onto the electrode and left to dry for 15 min at 40 °C. The electrode was covered with a Nafion film by drop-casting 30 µL of Nafion (1.0 wt.%) in isopropyl alcohol and dried at 40 °C for 10 min. 

### 2.6. Electrochemical Detection of Glucose on CQDs/Cu_2_O-Modified GCE

Electrochemical experiments were performed using an Autolab potentiostat 20 (Eco Chemie, Utrecht, The Netherlands). An Ag/AgCl (Bioanalytical Systems, Inc., West Lafayette, IN, USA) electrode was used as reference electrode, and platinum wire as counter electrode. Cyclic voltammetry (CV) measurements were performed in aqueous solutions of 0.1 M NaOH in the absence and presence of glucose. 

Chronoamperometric detection of glucose on the CQDs/Cu_2_O-modified GCE (3 mm in diameter) was performed in stirring alkaline solution (0.1 M NaOH) by applying a constant potential of +0.55 V to the working electrode. When the background current became stable (after 100 s), a glucose solution was subsequently added and the current was then measured.

### 2.7. Determination of Glucose Content in Human Serum Using a Colorimetric Method

First, a standard calibration curve for glucose was obtained by mixing a freshly prepared phenolic solution (1 mL; 5 wt.%) with 1 mL aliquot of glucose solution at different concentrations. Concentrated sulphuric acid (5 mL) was rapidly added to the mixture and left for 20 min under shaking before UV/Vis absorption spectra were recorded at 490 nm. Blank solutions were prepared in an identical manner, except that no glucose was added. Human serum (50 μL) was diluted five times (volume final: 250 µL) in water and mixed with 250 μL freshly prepared phenolic solution (1 mL; 5 wt.%). Concentrated sulphuric acid (1.25 mL) was added, and the mixture was left for 20 min under shaking before a UV/Vis spectrum of the serum sample was recorded. 

### 2.8. Characterization

Powder X-ray diffraction (XRD) patterns were collected on a Bruker D8 advance diffractometer (Cu-Kα radiation, 1.54056 Å, Bruker, Billerica, MA, USA) with an applied voltage of 40 kV and an anode current of 40 mA in the *2θ* range of 10°–80°.

Transmission electron microscopy (TEM) imaging was performed on a FEI Tecnai G2-F20 microscope operating (FEI, Hillsboro, OR, USA) at an accelerating voltage of 200 kV. 

Micro-Raman spectroscopy measurements were performed on a Horiba Jobin Yvon LabRam HR micro-Raman system combined with a 473 nm (1 mW) laser diode as excitation source. Visible light was focused by a 100× objective. The scattered light was collected by the same objective in backscattering configuration, dispersed by a 1800 mm focal length monochromator and detected by a charge-coupled device (CCD) camera.

UV/Vis absorption spectra were recorded using a Perkin Elmer Lambda UV/Vis 950 spectrophotometer (Perkin Elmer, Waltham, MA, USA) in quartz cuvettes with an optical path of 10 mm. The wavelength range was 450–550 nm. 

Thermogravimetric analysis (TGA) measurements were carried out in Al_2_O_3_ crucibles in a nitrogen atmosphere at a heating rate of 10 °C/min using a TA Instruments Q50 thermogravimetric analyzer.

X-ray photoelectron spectroscopy (XPS) experiments were performed in a PHl 5000 VersaProbe-Scanning ESCA Microprobe (ULVAC-PHI, Chigasaki, Japan) instrument at a base pressure below 5 × 10^−9^ mbar. Monochromatic AlKα radiation was used, and the X-ray beam, focused to a diameter of 100 µm, was scanned on a 250 × 250 µm surface at an operating power of 25 W (15 kV). Photoelectron survey spectra were acquired using a hemispherical analyzer at pass energy of 117.4 eV with a 0.4 eV energy step. Core-level spectra were acquired at a pass energy of 23.5 eV with a 0.1 eV energy step. All spectra were acquired at 90° between X-ray source and analyzer and with the use of low energy electrons and low energy argon ions for charge neutralization. After subtraction of the Shirley-type background, the core-level spectra were decomposed into their components with mixed Gaussian–Lorentzian (30:70) shape lines using the CasaXPS software. Quantification calculations were performed using sensitivity factors supplied by PHI. The sample was prepared by casting a concentrated aqueous solution of the material onto silicon substrate followed by drying at 100 °C for 1 h.

## 3. Results

### 3.1. Fabrication of CQDs/Cu_2_O Electrocatalytic Electrodes for Glucose Sensing

CQDs were synthesized at 50 °C via the hydrothermal carbonization method, using fructose as carbon source [18,19]. The obtained CQDs aqueous solution (1 mg/mL) was mixed with copper perchlorate. Upon heating to 80 °C for 3 h, Cu_2_O nanostructures were successfully deposited on the CQDs surface. 

**Figure 1:**
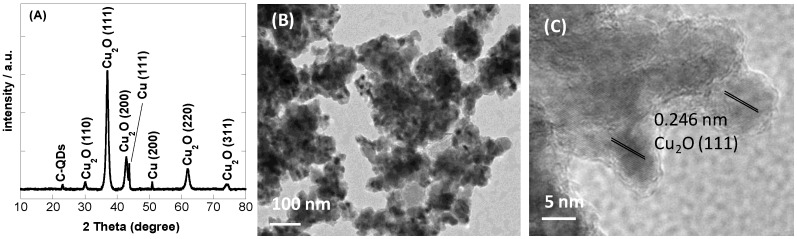
Characterization of carbon quantum dots loaded with copper oxide nanoparticles (CQDs/Cu_2_O NPs): (**A**) X-ray powder diffraction (XRD) pattern; (**B**) transmission electron microscopy (TEM) image; (**C**) high-resolution TEM (HRTEM) image.

Figure 1A depicts the XRD pattern of the as-obtained nanocomposite. Some broad and sharp peaks appeared simultaneously, which were identified to be the reflections of at least two different crystalline phases of Cu (ICDD No. 65-9026) and Cu_2_O (ICDD No. 78-2076). The corresponding reflection planes of different phases were 43.39° and 50.67° for Cu(111) and Cu(200), respectively, and 29.5°, 36.4°, 42.3°, 61.6°, and 73.8° for Cu_2_O(110), Cu_2_O(111), Cu_2_O(200), Cu_2_O(220), and Cu_2_O(311), respectively. The results suggest that the copper nanoparticles deposited under our experimental conditions consist mostly of copper oxide. In addition, a small peak at ~22.9° was observed and assigned to the presence of carbon from the CQDs. The results are in accordance with recently reported data on reduced graphene oxide modified with Cu_2_O [20], and Cu_2_O-modified multi-walled carbon nanotube nanostructures [21] and CQDs [17]. 

A low-magnification TEM image (Figure 1B) of the CQDs/Cu_2_O nanocomposite indicates that the particles are slightly aggregated. In accordance with XRD analysis, the high-resolution TEM image (Figure 1C) identified the presence of the Cu_2_O nanostructures with the lattice fringes of the (111) plane of the Cu_2_O with an interplanar spacing of 0.246 nm.

**Figure 2:**
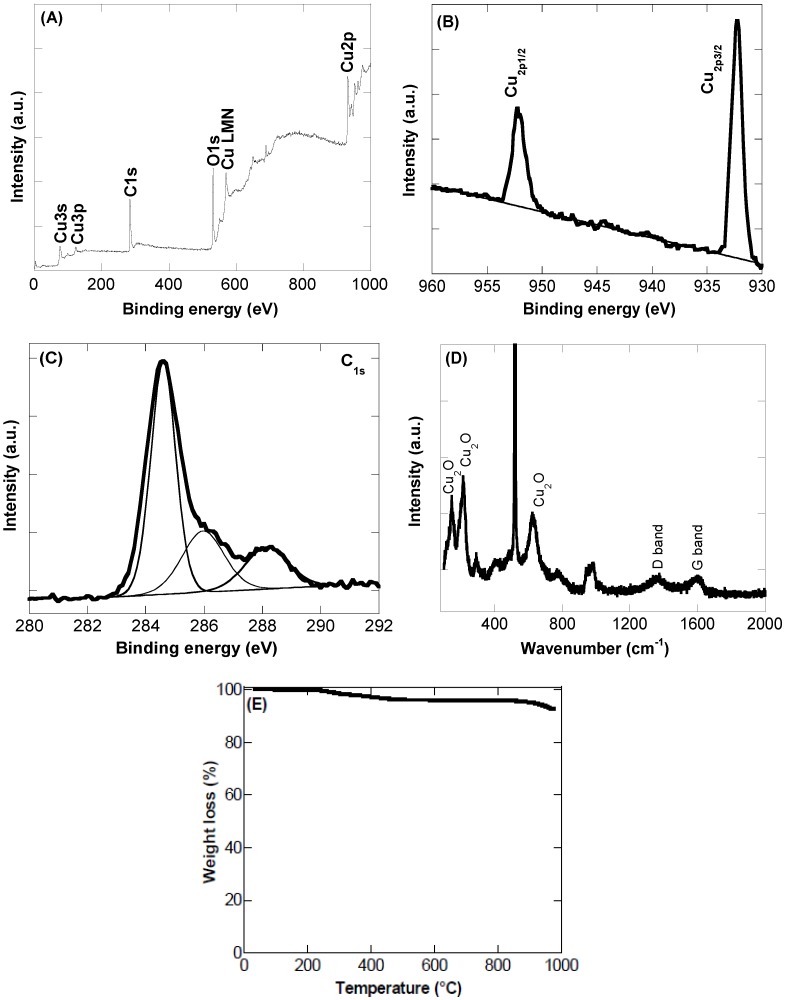
(**A**) X-ray photoelectron spectroscopy (XPS) survey spectrum; (**B**) Cu_2p_ core level spectrum; (**C**) C_1s_ core level spectrum; (**D**) Raman spectrum; (**E**) thermogravimetric analysis (TGA) thermogram of CQDs/Cu_2_O nanocomposite.

XPS analysis was further used to obtain more insight into the chemical composition of the as-synthesized CQDs/Cu_2_O nanocomposite. The XPS survey spectrum shows bands at 285 eV (C_1s_), 531 eV (O_1s_), and bands linked to Cu at 77 eV (Cu_3s_), 123 eV (Cu_3p_), and 932 eV (Cu_2p_) (Figure 2A) with an overall Cu content of 4.2 at%. The high-resolution XPS spectrum of Cu_2p_ (Figure 2B) reveals the presence of peaks at 932.7 and 952.6 eV attributed to Cu_2p3/2_ and Cu_2p1/2_, respectively, from Cu_2_O. 

The high resolution spectrum of C_1s_ (Figure 2C) exhibits a main peak at 283.6 eV attributed to the C–C bond with sp^2^ orbital configuration, and further bands at 285.1 eV and 287.3 eV assigned to sp^3^ hybridized carbons and C=O functions, respectively.

The CQDs/Cu_2_O was in addition studied by Raman spectroscopy (Figure 2D). Several Raman-active modes are observed. The peaks at 148, 216, and 622 cm^−1^ are characteristic of Cu_2_O [22]. The presence of CQDs is indicated by the bands at 1356 cm^−1^ and 1582 cm^−1^, originating from the G and D band contributions of the CQDs, respectively, due to the stretching motion of sp^2^ carbons and from defects in the hexagonal sp^2^ carbon network. 

To gain more insight into the thermal stability of the CQDs/Cu_2_O nanocomposite, thermogravimetric analysis was performed (Figure 2E). The TGA thermogram shows a small weight loss below 100 °C due to the desorption of water molecules with a further weight loss of ≈4% between 200–500 °C attributed to the removal of oxygen-containing functional groups on CQDs. No significant weight loss was observed above 500 °C, indicating the good thermal stability of the nanocomposite.

### 3.2. Non-Enzymatic Glucose Sensing

**Figure 3:**
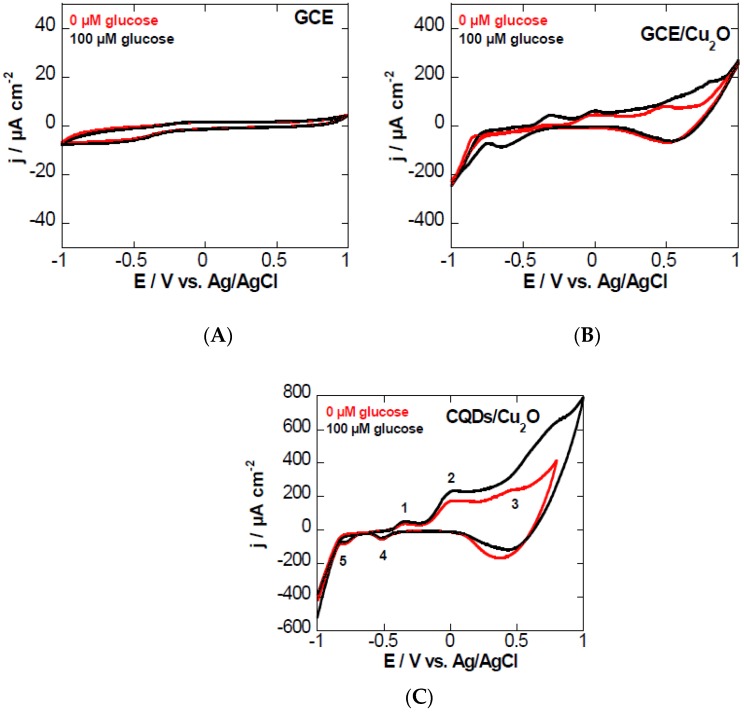
Cyclic voltammograms of (**A**) glassy carbon electrode (GCE); (**B**) GCE/Cu_2_O; and (**C**) GCE modified with CQDs/Cu_2_O by drop casting in 0.1 M NaOH aqueous solution in the absence (red) and presence of glucose (black, 100 µM).

The electrochemical behavior of a GCE (Figure 3A), a GCE modified with Cu_2_O (Figure 3B), and a GCE modified with CQDs/Cu_2_O (Figure 3C) in 0.1 M NaOH aqueous solution in the absence of glucose was evaluated using cyclic voltammetry (CV). The GCE and the GCE/Cu_2_O electrodes showed no or low electrocatalytic activity for glucose oxidation. The CV of the GCE modified with CQDs/Cu_2_O comprises several redox peaks. Peak 1 at −0.35 V corresponds to the transition of Cu0/Cu(I), while two processes are involved in peak 2 at ≈ −0.02V, ascribed to the transitions of Cu(0)/Cu(II) and Cu(I)/Cu(II), in accordance with other reports in the literature [17,23]. The formation of Cu(III) is initiated at around +0.53 V (peak 3). In the cathodic scan, peaks 4 and 5 at −0.51 V and −0.77 V correspond to the re-formation of Cu(I) and Cu(0), respectively.

In order to investigate the applicability of the CQDs/Cu_2_O-modified GC electrode for non-enzymatic glucose sensing, glucose was added to 0.1 M NaOH aqueous solution, and the voltammograms were recorded. A significant increase of the anodic current of peak 3 is obvious. The electrocatalytic oxidation peak is due to the conversation of Cu(III) to Cu(II) in basic medium. The Cu0/Cu(I) couple stays unchanged, ruling out the electrocatalytic reaction with glucose. The mechanism is believed to occur according to the equations below. This mechanism also highlights the importance of working under basic conditions. At neutral pH, no electrocatalysis takes place.

$$\begin{array}{l} \text{CQDs}/{\text{Cu}}_{2}O+{\text{OH}}^{-}\to \text{CQDs}/\text{CuOOH}+e^{-}\\ \text{CQDs}/{\text{Cu}}_{2}O+H_{2}O+2{\text{OH}}^{-}\to \text{CQDs}/\text{Cu}{{\left(\text{OH}\right)}_{4}}^{-}+e^{-}\\ {\text{Cu}}^{3+}+\text{glucose}+e^{-}\to {\text{Cu}}^{2+}+\text{gluconolactone} \end{array}$$

**Figure 4:**
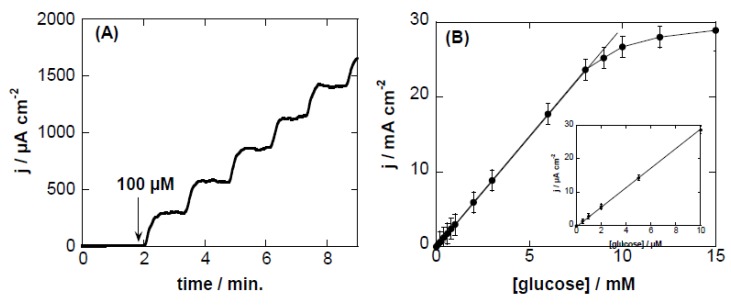
(**A**) Amperometric response curve of CQDs/Cu_2_O-modified GCE polarized at +0.55 V vs. Ag/AgCl upon successive additions of glucose (100 µM) in 0.1 M NaOH (up to a total of 500 µM); (**B**) Calibration curve for CQDs/Cu_2_O-modified GCE electrodes for the determination of glucose. The inset corresponds to a calibration curve for glucose concentrations of 0–10 µM.

The electrocatalytic response of the CQDs/Cu_2_O-modified GCE to glucose under basic conditions was further investigated by amperometric current–time response upon successive addition of different concentrations of glucose. Figure 4A displays the amperometric response of the modified electrode at an applied potential of +0.55 V vs. Ag/AgCl upon successive addition of glucose (100 µM) up to 500 µM. The oxidation current increased gradually upon the injection of increasing concentrations of glucose into the NaOH solution, and reached the maximum steady state current within ≈10 s. Figure 4B depicts the corresponding calibration curve of the current response versus glucose concentration. From the calibration curve, linearity (R^2^ = 0.999) over a wide concentration range (3 µM–8 mM) with a slope of 0.012 ± 2.95 mA·mM^−1^·cm^−2^ were determined. With a current noise of ≈0.4 µA, a detection limit of ≈6 µM was reached at a signal-to-noise ratio (S/N) = 3. The value is comparable to the detection limits reported for other glucose sensors based on electrochemical (EC) techniques (Table 1). However, the sensitivity of the sensor is considerably improved using this CQDs/Cu_2_O matrix. 

**Figure 5:**
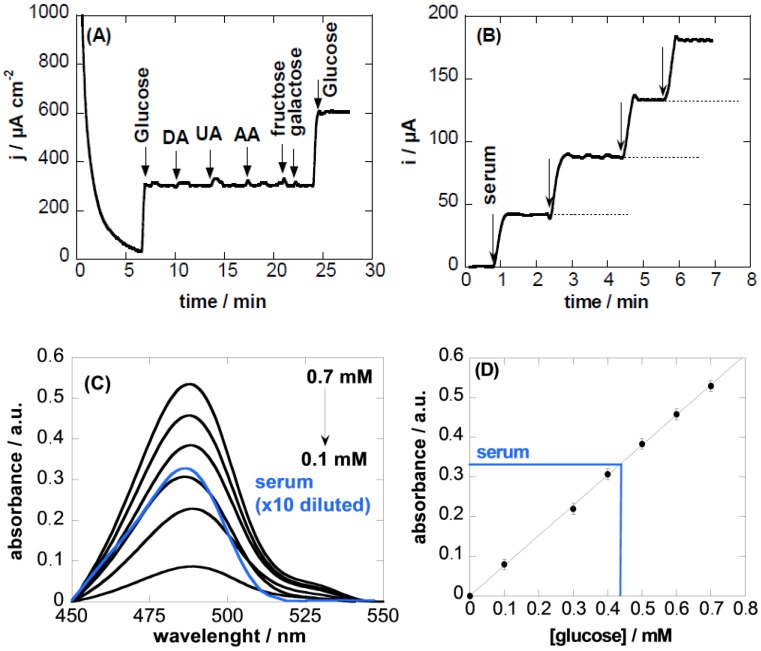
(**A**) Interference test of CQDs/Cu_2_O-modified GCE electrode in 0.1 M NaOH at +0.55 V with 100 µM glucose in the presence 500 µM dopamine (DA), uric acid (UA), ascorbic acid (AA), fructose, galactose; (**B**) current response to the addition of serum; (**C**) UV/Vis spectra of different concentrations of glucose as well as of the serum samples (ten times diluted) together with calibration curve (**D**).

To evaluate the effect of potential interfering compounds commonly found in biological fluids on the electrochemical detection of glucose, the current response of a glucose solution without and in the presence of ascorbic acid, uric acid, or dopamine of the same concentration was determined. To evaluate the selectivity towards glucose, other carbohydrate derivatives (such as fructose and galactose) were also examined. Figure 5A shows that no significant current increase was detected upon the addition of ascorbic acid, uric acid, or dopamine, suggesting that these species do not interfere with glucose detection under our experimental conditions. Similarly, no obvious response upon the addition of different interfering carbohydrate molecules was observed, indicating a good selectivity toward glucose detection.

The reproducibility of the electrode fabrication and use for glucose sensing is expressed in terms of relative standard deviation, which is found to be 5.3% at a glucose concentration of 1 mM. The long-term stability of the electrode was estimated after storage in a refrigerator at 4 °C for two months. The sensor retained about 90% of its initial current response to 1 mM glucose in 0.1 M NaOH aqueous solution at +0.55 V, suggesting a good stability of the electrode.

### 3.3. Sensing in Real Samples

A major concern inherent to any detection assay for biologically-relevant analytes in human serum is the possibility of background interferences. In order to evaluate the validity of the proposed glucose sensing electrode, serum samples of diabetic and healthy patients were analyzed with our sensor. Figure 5B shows the current response to 0.1 M NaOH (2 mL) upon the successive addition of undiluted serum. Using the calibration curve in Figure 4B, a glucose concentration of 4.2 ± 0.3 mM was determined. The concentration of glucose in human serum samples determined with the CQDs/Cu_2_O-modified GC electrode was compared to the concentration determined spectrophotometrically based on the phenol–sulfuric acid colorimetric method, well-established for analysis of carbohydrates. The UV/Vis absorption maximum obtained from the addition of 10 µL undiluted human serum solution into 1 mL phenol–sulfuric solution (10 times dilution) was compared to that of a calibration curve for glucose established under the same conditions (Figure 5C). The concentration of glucose in the human serum sample was determined to be 4.4 ± 0.3 mM. 

## 4. Conclusions

In summary, this work demonstrated the interest of CQDs/Cu_2_O-modified GC electrodes for sensing. The electrocatalytic activity of the material allowed for the detection of glucose in a non-enzymatic manner under basic conditions. While the obtained limit of detection is comparable to other carbon-based/Cu decorated nanomatrices, the CQDs/Cu_2_O matrix shows enhanced sensitivity and offers a facile and low-cost material for glucose sensing. Moreover, the utility of the sensors for the investigation of real samples was demonstrated upon the determination of glucose in the serum of patients. This work might open up new opportunities for the use of CQDs-based composites not only for fluorescence, but also for electrochemical detection of various analytes in complex media. 

## Figures and Tables

**Table 1:** Comparison of the analytical performance of other Cu-based non-enzymatic glucose sensors, as well as CQDs sensors. EC: electrochemical; LOD: limit of detection; rGO: reduced graphene oxide; MWCNTs: multi-walled carbon nanotubes.

Sensing Material	Detection Method	Solution	Sensitivity (µA·µM^−1^·cm^−2^)	Linear Range (µM)	LOD (µM)	Reference
B-doped CQDs	fluorescence	Water (pH 7.4)	-	8–80	8	[11]
Boronic acid-CQDs	fluorescence	Water (pH 7.4)	-	0.1–20,000	100	[10]
Boronic-acid-CQDs	fluorescence	Water (pH 7.4)	-	9–900	1.5	[9]
Cu NPs/MWCNTs	EC	NaOH (20 mM)	0.27	10–300	0.5	[24]
Cu NPs/rGO	EC	NaOH (100 mM)	0.447	100–1200	3.4	[14]
Cu_2_O/SMWNTs	EC	NaOH (100 mM)	2.1	0.5–2500	0.2	[21]
Cu_2_O/CQDs	EC	NaOH (100 mM)	0.298	20–4300	8.4	[17]
Graphene wrapped Cu_2_O	EC	KOH (100 mM)	0.285	300–3300	3.3	[25]
CQDs/Cu_2_O	EC	0.1 M NaOH	2.95 ± 0.2	1.3–6000	6	This work
